# Fluorescence microscope light source based on integrated LED

**DOI:** 10.1038/s41377-023-01245-9

**Published:** 2023-09-12

**Authors:** Jianchen Zi, Hai Bi

**Affiliations:** Jihua Laboratory, No.28 Huandao South Road, Nanhai district, 528200 Foshan, China

**Keywords:** Integrated optics, Wide-field fluorescence microscopy

## Abstract

An LED-integrated excitation cube (LEC) was designed to address the limitations of conventional fluorescence lamps. The LEC has a decentralized structure, high optical power density, and efficient illumination. The optical efficiency of LECs is 1–2 orders of magnitude higher than that of mercury lamps, enabling high-quality fluorescence imaging with spectral coverage from UV to red. LECs can be easily installed on commercial fluorescence microscopes by replacing conventional fluorescence filter cubes, and a built-in LEC driver can identify the types of LEDs in different spectral bands to adopt optimal operating conditions.

## Abstract (web summary)

The LED-integrated excitation cube (LEC) is compact, low-cost, and easy to install, taking full advantage of the benefits of LED excitations, and can be used in any fluorescent microscope.

## Graphical abstract

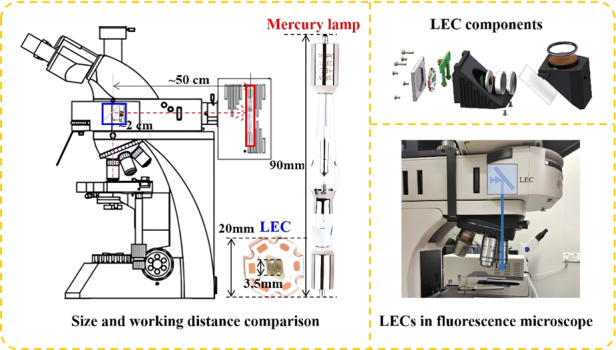

Fluorescence microscopy, which includes the latest advancements in super-resolution microscopy^1^, has become an important tool for biological research. The use of a wide range of fluorescent probes and light sources is crucial for imaging multiple subcellular structures and biomolecules simultaneously. There are various commercially available organic and inorganic fluorescent probes, including fluorescent proteins that emit in all spectral regions^2–7^. However, conventional light sources, such as mercury, xenon, and halogen lamps, suffer from problems of short service life, low power density, massive heat production and high optical efficiency loss. Therefore, light sources have evolved from conventional lamps to energy-efficient and solid-state light sources like lasers and LEDs^8^.

LED technology has rapidly developed in recent years, becoming a popular choice for illumination in various fields. LEDs have several advantages over traditional light sources, including high luminous efficiency, small size, low power consumption, long operational lifetime (around 10000–15000 h), and low price^9^. In addition, LEDs cover all wavelengths from UV to IR, providing flexibility for multiplexed biomedical imaging applications^10,11^. The LED industry has also grown significantly, resulting in increased production, reduced costs, and the development of new materials with the potential for solid-state illumination applications^12–15^. Overall, the continued development and advancement of LED technology have made it a promising and widely used illumination source in various fields.

A recent work^16^ in *Light: Advanced Manufacturing* by Liu and collaborators from the Southern University of Science and Technology and University of Technology Sydney introduces a new LED fluorescence light source called the LED-integrated excitation cube (LEC), as shown in Fig. 1. This design can be used in any fluorescent microscope and includes electronics built into the 6-position filter cube turret (8-position in the latest design) to enable independent control of each excitation channel. The LEC is compact, low-cost, and easy to install, taking full advantage of the benefits of LED excitations. Additionally, by pulsing the supply current, the LEC can provide high optical power density with reduced phototoxicity. When synchronized with a time-gated detection unit, LEC-powered microscopes can be used for time-gated luminescence imaging applications.

**Fig. 1:**
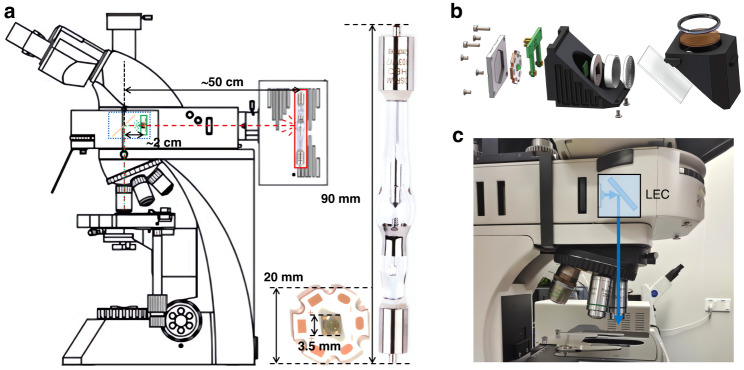
LEC integrated components. **a** The size and working distance comparison between LED of LEC and mercury lamp. LEC is mounted inside a microscope filter cube with the excitation light collimated by a Fresnel lens and reflected by a dichroic mirror into the objective lens. **b** The assembly of LEC components designed for fluorescence microscope. **c** LECs were mounted in fluorescence microscope

This work provides a new solution for the field of fluorescence microscopy by integrating LED, filters, Fresnel lenses, and dichroic mirrors into a filter cube, achieving the advantages of selectable excitation wavelengths, small size, low cost, and long lifetime. Simulation analysis shows that using a Fresnel lens can optimize the excitation delivery efficiency and optical power density of the specimen, limiting the optimal mounting distance to 15 mm. LECs can be easily installed in commercial microscopes, and experiments have shown that they can achieve widefield fluorescence imaging across a broad range of excitation spectra. LECs also offer higher excitation power for wavelengths beyond 600 nm, resulting in enhanced contrast in fluorescence imaging compared to traditional mercury lamps. The LED’s voltage, current, and switching frequency can also be adjusted for time-gated luminescence microscopy. Through simulation analysis and experimental verification, this scheme has excellent fluorescence imaging performance and achieves high-contrast fluorescence microscopy imaging in a wide wavelength range. Additionally, this scheme can also perform time-gated fluorescence microscopy imaging and has broad application prospects. Therefore, this work has high research value and application potential.
